# Genotyping-by-Sequencing Identifies Historical Breeding Stages of the Recently Domesticated American Cranberry

**DOI:** 10.3389/fpls.2020.607770

**Published:** 2020-12-16

**Authors:** Luis Diaz-Garcia, Giovanny Covarrubias-Pazaran, Jennifer Johnson-Cicalese, Nicholi Vorsa, Juan Zalapa

**Affiliations:** ^1^Instituto Nacional de Investigaciones Forestales, Agrícolas y Pecuarias (INIFAP), Aguascalientes, Mexico; ^2^Centro Internacional de Mejoramiento de Maíz y Trigo (CIMMYT), El Batan, Mexico; ^3^Marucci Center for Blueberry and Cranberry Research and Extension Center, Rutgers University, Chatsworth, NJ, United States; ^4^Department of Plant Science, Rutgers University, New Brunswick, NJ, United States; ^5^Department of Horticulture, University of Wisconsin, Madison, WI, United States; ^6^USDA-ARS, Vegetable Crops Research Unit, University of Wisconsin, Madison, WI, United States

**Keywords:** American cranberry, domestication, genetic diversity, genome-wide association mapping, linkage disequilibrium, population structure, *Vaccinium*

## Abstract

The cranberry (*Vaccinium macrocarpon* Ait.) is a North American fruit crop domesticated less than 200 years ago. The USDA began the first cranberry breeding program in response to false-blossom disease in 1929, but after the first generation of cultivars were released in the 1950s, the program was discontinued. Decades later, renewed efforts for breeding cranberry cultivars at Rutgers University and the University of Wisconsin yielded the first modern cultivars in the 2000’s. Phenotypic data suggests that current cultivars have changed significantly in terms of fruiting habits compared to original selections from endemic populations. However, due to the few breeding and selection cycles and short domestication period of the crop, it is unclear how much cultivated germplasm differs genetically from wild selections. Moreover, the extent to which selection for agricultural superior traits has shaped the genetic and phenotypic variation of cranberry remains mostly obscure. Here, a historical collection composed of 362 accessions, spanning wild germplasm, first-, second-, and third-generation selection cycles was studied to provide a window into the breeding and domestication history of cranberry. Genome-wide sequence variation of more than 20,000 loci showed directional selection across the stages of cranberry domestication and breeding. Diversity analysis and population structure revealed a partially defined progressive bottleneck when transitioning from early domestication stages to current cranberry forms. Additionally, breeding cycles correlated with phenotypic variation for yield-related traits and anthocyanin accumulation, but not for other fruit metabolites. Particularly, average fruit weight, yield, and anthocyanin content, which were common target traits during early selection attempts, increased dramatically in second- and third-generation cycle cultivars, whereas other fruit quality traits such as Brix and acids showed comparable variation among all breeding stages. Genome-wide association mapping in this diversity panel allowed us to identify marker-trait associations for average fruit weight and fruit rot, which are two traits of great agronomic relevance today and could be further exploited to accelerate cranberry genetic improvement. This study constitutes the first genome-wide analysis of cranberry genetic diversity, which explored how the recurrent use of wild germplasm and first-generation selections into cultivar development have shaped the evolutionary history of this crop species.

## Introduction

The American cranberry (*Vaccinium macrocarpon* Ait.) is a woody temperate perennial fruit crop native to the acidic bogs of North America (Vander Kloet, 1988). The species is especially important because of its high polyphenolic based nutritional benefits and economic value in the United States and internationally (Neto, 2007). Cranberry production has increased considerably during the last few decades, primarily due to the adoption of recently bred highly productive cultivars, acreage expansion, implementation of improved agronomic practices, e.g., especially plant nutrition and in response to the diversification of cranberry products such as the development of sweet and dried cranberries or SDCs (Gallardo et al., 2018; Vorsa and Zalapa, 2020).

Cranberry domestication and culture began in the 1820’s with the selection of the first cranberry varieties from native populations in Massachusetts, “Howes” and “Early Black,” cultivars which were widely planted commercially in 1850’s (Stevens et al., 1957; Chandler and Demoranville, 1958; Eck, 1990; Vorsa and Zalapa, 2020). From the late 1850’s to early 1900’s, hundreds of varieties were selected from native populations (Eck, 1990). Some of these variates, such as “the Big Four” (“Early Black,” “Howes,” “McFarlin,” and “Searles”), which are all native selections, were used commercially for many decades during the cranberry industry expansion (Eck, 1990; Vorsa and Zalapa, 2020). In response to “false blossom” phytoplasma disease, the first cranberry breeding program was initiated by the USDA in the late 1920’s. The progeny from the initial crosses, which were product of selection and controlled breeding, were planted at Whitesbog, New Jersey, having high “false-blossom” disease pressure. Selections were evaluated by a “preference test” to the blunt-nosed leaf hopper, the vector for false-blossom. Cultivars initially released derived from early breeding programs included six varieties, “Stevens,” “Beckwith,” and “Wilcox” released in 1950, and “Pilgrim,” “Bergman,” and “Franklin” in the early 1960’s. “Bergman,” “Franklin,” “Pilgrim,” and “Wilcox” were considered to have resistance to feeding by the bunt-nosed leaf-hopper (Dana, 1983). Other varieties with improved fruit quality were release during the early 2000’s, including “HyRed,” the first cranberry modern cultivar, and “Mullica Queen,” the most popular new hybrid today (Gallardo et al., 2018; Vorsa and Zalapa, 2020). Advanced crosses and selections have been derived from native and elite materials and are currently under phenotypic evaluation mainly in New Jersey, Massachusetts, Wisconsin and Oregon. Noteworthy, most of the cranberry production is currently based on a few cultivars, including “Stevens” (40% of all acreage worldwide), which is not considered highly resistant to blunt-nosed leafhopper feeding, and “Mullica Queen” (Gallardo et al., 2018; Vorsa and Zalapa, 2020). However, to some extent, some growers still use native selections made in the early 1800’s, e.g., “Ben Lear,” “Howes,” “Early Black,” and “McFarlin,” among others (Vorsa and Zalapa, 2020).

The development of cranberry marker-based breeding strategies has been virtually non-existent, mainly because molecular tools were not available until 2010, with the advent of the first molecular maps and other sequencing efforts (Vorsa and Zalapa, 2020). Cranberry genetics studies that have contributed to the ongoing development of marker-breeding strategies include a genome assembly (Polashock et al., 2014), several high-density linkage maps (Schlautman et al., 2015; Covarrubias-Pazaran et al., 2016; Schlautman et al., 2017a), QTL mapping of fruit rot resistance (Georgi et al., 2013; Daverdin et al., 2017), fruit size and shape (Diaz-Garcia et al., 2018a), and fruit metabolites (Diaz-Garcia et al., 2018b; Fong et al., 2020), and genomic selection studies (Covarrubias-Pazaran et al., 2018). Additionally, only a few studies have investigated the genetic diversity in cranberry cultivars, cranberry wild populations, and native populations (Fajardo et al., 2013; Zalapa et al., 2015; Schlautman et al., 2017b; Schlautman et al., 2018; Rodríguez-Bonilla et al., 2019), most of them based on a limited number of genetic markers.

The evolutionary and genetic history of cranberry is little known. Cranberry is a very interesting fruit crop model having both asexual and sexual reproduction, overlapping generations, a limited natural range in North America, and a recent domestication history. These characteristics have impacted the selection of favorable alleles, admixture, and introgression during the breeding process, as well as distant natural selection events such as severe glacial bottlenecks during the Pleistocene (Bruederle et al., 1996) and reproductive adaptations such clonality and selfing fertility, (Vander Kloet, 1988; Eck, 1990; Vorsa and Zalapa, 2020). To gain a better understanding of the evolutionary, domestication, and breeding history of cranberry, we investigated genome-wide variation patterns as well as the genetic basis of horticultural traits possibly targeted during the initial process of domestication. Our study was conducted using 362 diverse cranberry materials, which included cultivars derived from breeding programs, advanced selections, native domesticated clones, and germplasm directly collected from native populations. This is the first study aiming to understand the domestication and breeding history of this North American native crop by examining the genetic diversity and population structure in a wide set of cranberry materials spanning wild, intermediate, and elite breeding materials, as well as current cultivars.

## Materials and Methods

### Germplasm Composition

The cranberry diversity panel studied here included 362 accessions (Table 1). From these, 121 were clones collected directly from native populations (WLD) and 111 were native selections (NS) used commercially in the past (some still used today) or that have contributed to genetic enhancement of this crop; therefore, NS accessions represent the first-generation cranberry selections. The WLD accessions were originally collected by Leo Bruederle and Nick Vorsa (Bruederle et al., 1996). The sampling was done randomly in the wild to cover the known range of cranberry in the United States. While populations were sampled throughout much of the range of this species, *V. macrocarpon* is restricted to relatively small, disjunct, very specific environments in MI, WI, MA, PA, NJ, DE. There was no particular selection or preference in the selection of the population or for any phenotypic traits. Additionally, our diversity panel included 26 cultivars/hybrids (CLT1) that correspond to second-generation selections, or first-generation hybrids, as well as 104 advanced selections (CLT2) derived from current breeding programs and that represent third-generation selections. This cranberry diversity panel is the largest and most complete collection of cranberry genotypes in the world and is currently housed at Rutgers University, Marucci Center for Blueberry and Cranberry Research and Extension Center in Chatsworth, NJ. Accession origin, a geographical infomap, pedigree, and additional information regarding these 362 accessions is provided in Supplementary Table 1 and Supplementary Figure 1.

**TABLE 1:** Origin of the cranberry accessions included in this study, grouped by subgroup.

Location	CLT1	CLT2	NS	WLD	Total
Not assigned	26	104	0	0	130
Canada	0	0	1	4	5
DE, United States	0	0	0	8	8
MA, United States	0	0	37	44	81
ME, United States	0	0	1	3	4
MI, United States	0	0	1	9	10
NJ, United States	0	0	21	14	35
NY, United States	0	0	0	20	20
OR, United States	0	0	7	0	7
PA, United States	0	0	0	7	7
WA, United States	0	0	6	0	6
WI, United States	0	0	37	4	41
WV, United States	0	0	0	8	8
Total	26	104	111	121	362

*CLT1, CLT2, NS, and WLD correspond to cultivars/hybrids, advanced selection, native selections, and wild/native subgroups, respectively. CLT1 and CLT2 subgroups do not have an assigned location.*

### Genotyping-by-Sequencing

Genotyping was performed using genotyping-by-sequencing (GBS), following Covarrubias-Pazaran et al. (2016). EcoT221 digested DNA was sequenced on the Illumina HiSeq 2000 sequencing platform (Illumina, San Diego, CA, United States), at the Genomic Diversity Facility in Cornell University (Ithaca, NY, United States). TASSEL (Bradbury et al., 2007) was used for single nucleotide polymorphism (SNP) calling (quality filter >20) using an unreleased cranberry genome assembly as reference. This genome assembly includes 12 pseudomolecules of size 33–50 Mb. Variant calling file was read on R for further processing. SNP markers with excessive missing data (>30%) and minor allele frequency (MAF) <0.05 were removed.

### Diversity Panel Phenotyping

The cranberry diversity panel was phenotyped for fruit yield, average fruit weight, fruit rot, total anthocyanin content (TACy), Brix degrees, and acids during 2006–2010 and 2014–2015 at Rutgers University. Total fruit yield per area was calculated by harvesting and weighing all the fruit within 0.09 m^2^; from that, 100 fruit were randomly selected and weighed to calculate average fruit weight, as described in Georgi et al. (2013) and Covarrubias-Pazaran et al. (2016). TACy, Brix degrees and acids were quantified as in Diaz-Garcia et al. (2018b). Briefly, TACy was measured spectrophotometrically under acidic conditions at 515 nm; Brix degrees was quantified with a refractometer as percent of soluble solids, and acids (expressed as milliequivalents of citric acid) using 0.1 N NaOH and an endpoint of pH 8.1. Genomic best linear unbiased predictors (GBLUPs) were computed for each accession using linear mixed models in sommer (Covarrubias-Pazaran, 2016). Spatial variation was modeled using a 2-dimensional spline. All components in the model were considered as random.

### Population Structure and Diversity

Population structure of the 362 cranberry accessions was assessed using a Structure-like population genetic analysis with the R package LEA (Frichot and François, 2015). We tested different number of clusters (*K*), from 2 to 10 (with five replicates each), and selected a *K* value using a cross-entropy criterion computed by the package. Moreover, we performed hierarchical clustering using the Ward clustering algorithm implemented in the *hclust* function in R, as in Siol et al. (2017), which was further visualized in a tree and compared with the Structure population analysis.

Genetic relationship among accessions were also investigated using principal component analysis (PCA) in R. The complete SNP dataset was converted into a numeric format (−1, 0, and 1) using sommer (Covarrubias-Pazaran, 2016), and then, inputted into the *prcomp* R function. Principal components 1–10 were used in further analysis.

### Fixation Index (*F*_ST_) Scans

Following our analysis of population structure and genetic diversity, we investigated which regions of the genome were the most divergent between the different cranberry subgroups studied here. For that purpose, we computed genome-wide fixation index (*F*_ST_) values between all subgroup pairs using the pegas package (Paradis, 2010).

### Estimation of Linkage Disequilibrium and Genome-Wide Association Mapping

Pairwise linkage disequilibrium (LD) explained by pairwise correlation coefficient (*r*^2^) was computed per chromosome using non-overlapping windows of 10 Kb length. The intra-chromosomal genome-wide linkage disequilibrium decay rate was estimated separately for all four subgroups. Linkage disequilibrium calculations were made using in-house R scripts. Association mapping was conducted for all phenotypic traits using mixed linear models, where PC1-10 from the PCA analysis, population structure probabilities, and an additive relationship matrix computed with the *A.mat* function of the rrBLLUP package (Endelman, 2011) were included. Mixed linear models were solved using the GWAS function in sommer (Covarrubias-Pazaran, 2016).

## Results

### GBS Sequencing

**FIGURE 1:**
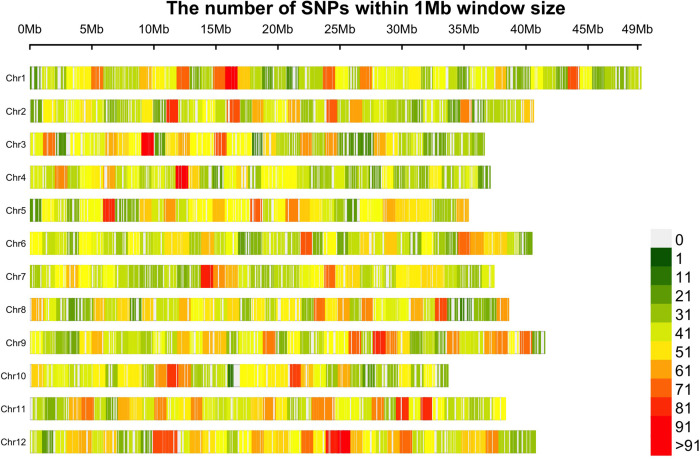
The distribution of 21,179 SNP markers in the twelve cranberry (*Vaccinium macrocarpon* Ait.) chromosomes. Marker counts are based on 1 Mb non-overlapping windows. Figure made with the R package CMplot.

A collection of 362 cranberry accessions representing a wideset of wild and cultivated diversity was characterized using GBS. In total, 110671 non-filtered SNPs were obtained from more than 1.2 billion reads assigned to 6.17 million tags (minimum read per tag = 3). Then, 21,179 SNPs (average site depth was 23.16 reads) uniquely assigned to a genomic position were finally selected after filtering for missing data and MAF. An average of 1764.20 SNP markers were obtained per chromosome (chromosome 1 was the one with the most markers, 2145, and chromosome 10 with the fewest, 1504). The average gap between SNP markers was 22.19 Kb (Figure 1). Polymorphic SNPs among CLT2 (19485), CLT1 (18986), NS (20623), and WLD (20394) subgroups represented 92.0, 89.6, 97.4, and 96.3%, respectively, which is common pattern consistent with progressive bottlenecks on genetic diversity during domestication and further genetic breeding (Varshney et al., 2017).

The distribution of MAF across markers was fairly consistent across subgroups. A large portion of markers showed MAFs <0.1 (Supplementary Figure 2). In particular, native populations (WLD) and native selections (NS) showed more clearly this pattern, compared with CTL1 and CLT2 that showed a smother distribution. Inspection of joint MAF distributions (i.e., two-way contingency tables between pairs of subgroups) showed similarities between NS and WLD, and CLT1 and CLT2, which are subsequent steps in the domestication/breeding processes. Furthermore, NS and CLT1 showed moderately similar joint MAF distributions. Conversely, a WLD and CLT1/CLT2 showed very dissimilar MAF distributions, which is expected because these subgroups represent the start and end points of the domestication/breeding process.

### Population Structure and Genetic Diversity

**FIGURE 2:**
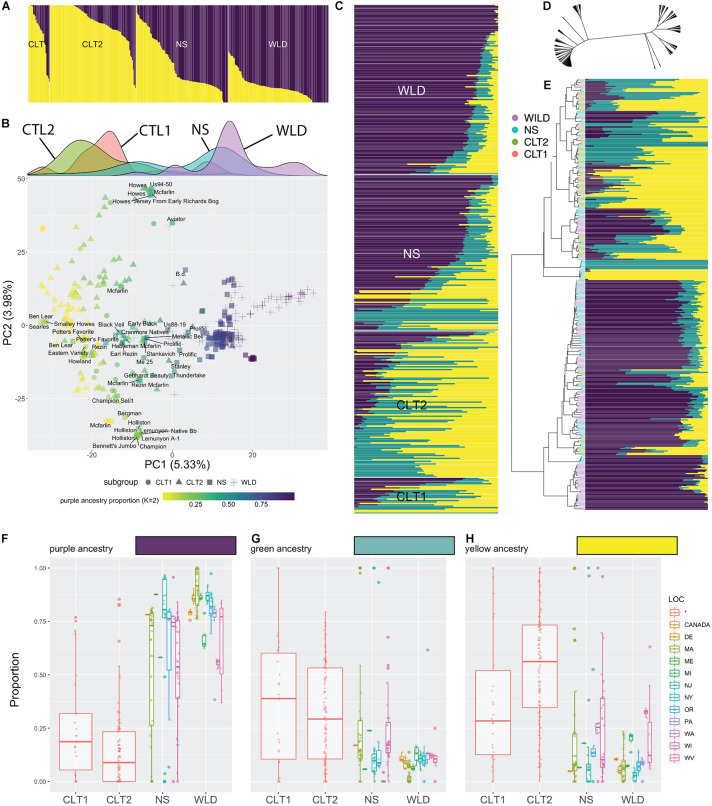
Admixture in cranberry (*Vaccinium macrocarpon* Ait.) subgroups resolved using 18,149 single nucleotide polymorphisms (SNP) scored on 362 accessions. **(A)** Results of population structure analysis, with *K* = 2; subgroups CLT1 (cultivars), CLT2 (advanced selections), NS (native selections), and WLD (native populations) are grouped. **(B)** Principal Component Analysis of 362 cranberry accessions scored with 21,177 SNP markers. Cranberry accessions are colored based on the purple ancestry proportion, when *K* = 2 (see panel **A**). Subgroups are represented as different marker symbols in the plot. Labels are shown only for NS accessions when PC1 <5. Density plots per subgroup among PC1 are shown in the top. **(C)** Results of population structure analysis, with *K* = 3, organized by subgroup. **(D)** Reticulated tree including the 362 accessions based hierarchical clustering (Ward’s minimum variance method). **(E)** Reorganization of population structure results (*K* = 3) based on hierarchical clustering. Proportion of each of the three ancestries (*K* = 3), purple **(F)**, green **(G)**, and yellow **(H)** across subgroups and their geographical origin.

Based on population structure analysis, we observed a continuous distribution of ancestry levels across all cranberry accessions (Figure 2). Our analysis differentiated WLD, NS, CLT1, and CTL2, however, we did not observe a clearly dominant ancestry among the subgroups. When *K* = 2 (Figure 2A and Supplementary Table 2), we found an increasing proportion of the yellow ancestry (and decreasing proportion of purple ancestry) as we transition from WLD to CLT2. Particularly, most of the WLD accessions had less than 25% of the yellow ancestry, whereas for most of the CLT2 and CTL1 accessions, the yellow ancestry proportion was at least 75%. PCA reflected a varying level of clustering of accessions belonging to the same subgroups (Figure 2B), similar to what was observed with the population structure analysis. For example, most of the WLD accessions tended to group well on the positive side of PC1 (5.33% explained variance), although some of them were more dispersed. Conversely, CLT2 and CTL1 accessions were mostly located on the negative side of PC1, whereas NS were dispersed in between. Occasionally, NS, CLT1, and CLT2 accessions formed small groups within the PC1 and PC2 (3.98% explained variance) space, particularly when CLT1 or CLT2 accessions have an NS origin. The density panel on Figure 2B (density plots per subgroup among PC1) clearly depicts the cranberry domestication and breeding history. Specifically, WLD and CLT1/CLT2 subgroups appeared on opposite extremes, whereas NS accessions, which represent first-generation selections are in between. Interestingly, NS density plot show a very wide distribution, with two apparent peaks. The peak toward the right side correspond mostly to accessions that have been continuously used to derive first (CLT1) and second-generation hybrids (CLT2).

Further partitioning of population structure results (*K* = 3, Figure 2C and Supplementary Table 3) showed that the purple ancestry remains as the main factor differentiating the four subgroups, similar to when *K* = 2 (Figure 2B). The yellow ancestry in *K* = 2 now is partitioned into yellow and green, and seems to set apart some accessions, especially in the NS subgroup. Hierarchical clustering (based on Ward’s minimum variance) suggested that the genetic relationships among accessions are highly reticulated (Figure 2D) and that there are two well defined groups, one mostly composed by CLT2 accessions, whereas the other was dominated by WLD accessions (although in both cases there was a considerable proportion of accessions belonging to both NS and CTL1 subgroups; Figure 2E). Reorganizing population structure data based on hierarchical clustering revealed that purple ancestry partially correlates with the two-group separation. Subsequent inspection of the level of ancestry by subgroup showed that CTL1 and CTL2 have considerably less proportion of the purple ancestry (Figure 2F), although accessions from other subgroups can also present low values. Green (Figure 2G) and yellow (Figure 2H) ancestries also varied among subgroups, but the differences were subtle.

Our data suggests a partial distinction between subgroups, this is, individuals in certain subgroups exhibit ancestry properties of another subgroup, presumably because of the recent history of cranberry domestication. Although CLT1 and CLT2 subgroups represent the current state of the cranberry germplasm availability, a large proportion of the immediate parental accessions of the cultivars/advanced selections used commercially belong to the NS subgroup, which is composed of first-generation selections from the wild. For example, out the 26 CLT1 cultivars studied here, 21 had an accession NS origin as a parent (at least one parent, some can have both). Similarly, out of the 121 advanced selections (CLT2), 51 had an accession of NS origin.

### Impact of Domestication and Breeding on Genetic Diversity

**FIGURE 3:**
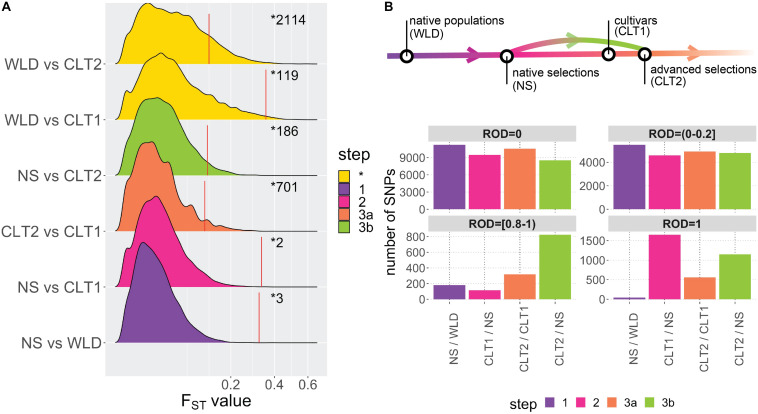
Impact of domestication and breeding on cranberry (*Vaccinium macrocarpon* Ait.) genetic diversity. **(A)** Density plots for *F*_ST_ values among all subgroup combinations. Colors are based on meaningful subgroup comparisons in the context of the cranberry domestication/breeding history (step 1 in red, step 2 in green, step 3a in blue, and step 3b in purple; non-categorized comparisons are displayed in yellow and as step *). Red vertical lines correspond to *F*_ST_ 95th percentiles (significant thresholds) obtained from permutation analysis on each subgroup comparison; numbers after the red line correspond to the number of significant *F*_ST_ values on each subgroup comparison. **(B)** Genomic regions with different levels of reduction of diversity (ROD = 0, ROD >0 and ≤0.2, ROD ≥0.8 and <1, and ROD = 1), organized according with A.

Using allele *F*_ST_ estimates (Supplementary Table 4), which measure the proportion of the variance in allele frequencies attributable to variation between groups, we investigated the divergence between the different cranberry subgroups included in this study (Figure 3A). Based on mean *F*_ST_’s among genome-wide estimates, maximum differentiation was computed for WLD vs. CLT2 (*F*_ST_ = 0.042), followed by WLD vs. CLT1 (*F*_ST_ = 0.034), NS vs. CLT2 (*F*_ST_ = 0.023), NS vs. WLD (F_ST_ = 0.013), NS vs. CLT1 (0.010), and CLT2 vs. CLT1 (0.007). Additionally, *F*_ST_ 95th percentiles, based 1000-iteration permutation tests (resampling memberships between two subgroups), were 0.35 (WLD vs. CLT1), 0.33 (NS vs. CLT1), 0.32 (CTL1 vs. CLT2), 0.12 (WLD vs. CLT2), 0.12 (NS vs. WLD) and 0.11 (NS vs. CLT2). Some genomic regions presented *F*_ST_ values considerably higher than *F*_ST_ 95th percentiles; for example, single SNP *F*_ST_ estimates were as a high as 0.594 (for WLD vs. CLT2), 0.550 (for WLD vs. CTL1), and 0.403 (for NS vs. CLT2).

To infer the effects of domestication and breeding on the genetic diversity of cranberry, we computed the reduction of diversity (ROD), based on the ratio of diversity between subgroups we assumed corresponded to the progression of domestication and breeding (Figure 3B). We defined native accessions (WLD) as the starting point in the domestication-breeding process. Then, native selections (NS) corresponded to first-generation selections from wild environments and that were used commercially; therefore, we defined NS accessions as the first step in the process (step 1 in Figure 3A). Subsequently, NS accessions served as a base for the development of CLT1 cultivars (step 2 in Figure 3A). Finally, CLT1 cultivars were used in advanced selections (CLT2, step 3a in Figure 3A). Occasionally, CTL2 advanced selections were derived from NS accessions, which would represent a two-step jump in the breeding process (step 3b in Figure 3A). ROD was estimated for pairs of subgroups comprising the steps commented above.

For maximum ROD (ROD = 1), there were 42 SNPs for step 1 (NS/WLD comparison), 1649 SNPs for step 2 (CLT1/NS comparison), 560 SNPs for step 3a (CLT2/CLT1 comparison), and 1152 SNPs for step 3b (CLT2/NS comparison). SNPs with ROD ≥0.8 and <1 were fewer than for ROD = 1. Steps 1, 2, 3a, and 3b had 180, 114, 318, and 823 SNPs, respectively. Finally, for low or null ROD (ROD >0 and ROD ≤0.2, or ROD = 0, respectively), most of the steps had consistently high number of SNPs and very few differences (Figure 3B).

### Linkage Disequilibrium and Genome-Wide Association

**FIGURE 4:**
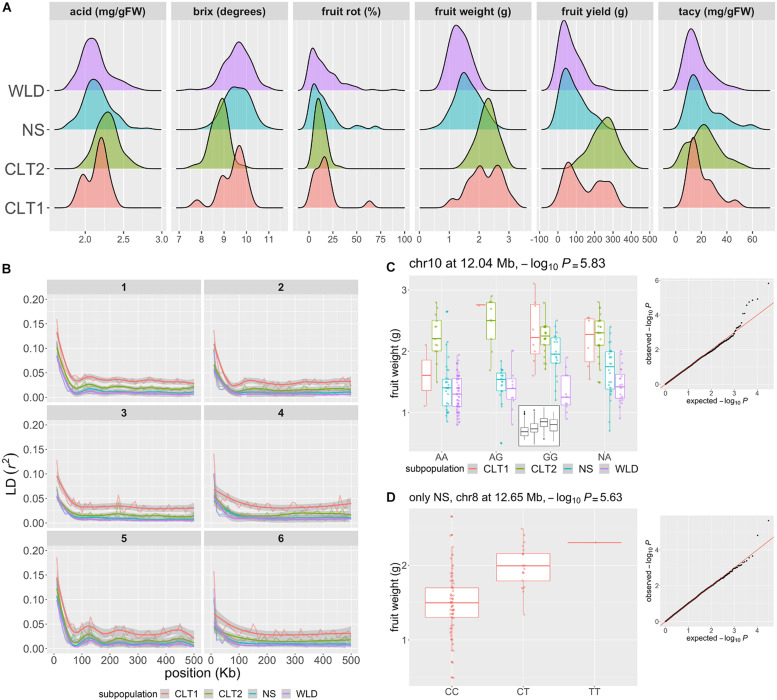
Genomic regions associated to phenotypic traits of horticultural interest in cranberry (*Vaccinium macrocarpon* Ait.). **(A)** Acid, Brix degrees, fruit rot, average fruit weight, fruit yield, and total anthocyanin content (TACy) variation across 293 accessions, colored by subgroup (WLD: native populations, NS: native selections, CLT2: advance selection, CLT1: cultivars/hybrids). **(B)** Patterns of intrachromosomal linkage disequilibrium (LD, *r*^2^) decay as a function of physical distance (only the first 500 kb are shown), colored by subgroup. LD decay was computed using 10 Kb bins. LD decay is shown for chromosomes 1–6 only; chromosomes 7–12 are presented in Supplementary Material. **(C)** Significant marker-trait association (MTA) for average fruit weight, with a marker in chromosome 10 position 12.04 Mb, using all 293 accessions and colored by subgroup. The –log_10_*P* for the MTA was 5.83 (significant at FDR = 0.1). In the small panel inside the main plot, boxplots are shown without separating by subgroup. **(D)** MTA for fruit weight, with a marker in chromosome 8 position 12.65 Mb, using only NS accessions (*n* = 97). The –log_10_*P* for the MTA was 5.63 (significant at FDR = 0.1).

We recorded average fruit weight, total fruit yield, fruit rot, TACy, Brix degrees, and acids for 282 accessions (97 WLD, 97 NS, 71 CLT2, and 17 CLT1) during 2006–2010 and 2014–2015 (Figure 4A and Supplementary Table 5). Fruit yield values ranged from 1.70 to 401.10 g (mean = 125.77 g); fruit weight from 0.50 to 3.10 g (mean = 1.72 g), fruit rot from 0 to 88.7% (mean = 14.49%), TACy from 2.00 to 62.00 mg/gFW (mean = 20.01 mg/gFW), Brix degrees from 7.50 to 11.00 (mean = 9.38), and acids from 1.78 to 2.81 mg/gFW (mean = 2.18 mg/gFW). We observed significant differences among subgroups for total fruit yield and average fruit weight (*P* = 0), TACy (*P* = 0.003), Brix degrees (*P* = 0), and acid (*P* = 7.149e-8). In contrast, we did not observe significant differences for fruit rot (*P* = 0.2735). Average fruit weight and total fruit yield were highly correlated (Pearson’s correlation, *r*^2^ = 0.70, *P* < 2.2e-16); Brix degrees were moderately correlated with both average fruit weight (*r*^2^ = −0.47, *P* < 2.2e-16) and total fruit yield (*r*^2^ = −0.43, *P* = 4.037e-14); fruit rot and TACy showed low correlation (*r*^2^ = 0.24, *P* = 4.988e-05), as well as acid and total fruit yield (*r*^2^ = 0.32, *P* = 2.234e-08) and acid and average fruit weight (*r*^2^ = 0.18, *P* = 0.002). Other trait comparisons were not significantly correlated (*P* = 0.05, Supplementary Table 6).

To estimate the linkage disequilibrium (LD) patterns in the different cranberry subgroups, we calculated *r*^2^ between all pairs of SNP markers (Figure 4B). Linkage disequilibrium decayed to its half-maximum within the first 20 Kb for all subgroups; however, further LD decay (after half-maximum value) was less marked for CLT1 compared to the rest of the subgroups; CLT2 showed a LD decay pattern similar to WLD and NS, presumably because the occasional use of NS accessions in CLT2 advanced selections. This LD decay pattern partially agrees with a strong selection bottleneck during the last stages of domestication/breeding.

To identify genomic regions associated with phenotypic variation, we performed GWAS using population structure (*K* = 3), principal components (PC 1-10), and kinship (additive relationship matrix) to account for population stratification. Using a threshold of FDR = 0.1, we identified one marker-trait association (MTA) for total fruit yield (chromosome 7) and five for average fruit weight (chromosomes 4, 5, 7, 9, and 10). Nevertheless, when we visually inspected each of these MTAs, we found that some of the associations may be spurious due to the high stratification of the subgroups utilized. Particularly, CLT1 and CTL2 accessions had considerably higher values for total fruit yield and average fruit weight than the other two subgroups, and the models used to account for population stratification may not allow the breaking down of this structure. For example, in Figure 4C, we show an MTA for average fruit weight in chromosome 10 at 12.04 Mb with −log_10_*P* = 5.83; as illustrated in the small boxplot inside the panel, the G allele seems to increase average fruit weight. However, when inspecting average fruit weight variation by both genotype and subgroup, we found that the significance of this association could be caused by a high number of WLD accessions with AA genotype, which in this case presented lower average fruit weight. Nevertheless, some these suspicious MTA for total yield and average fruit weight partially co-localized with QTLs previously identified using biparental populations, which may require further confirmation and testing (Georgi et al., 2013; Schlautman et al., 2015; Diaz-Garcia et al., 2018a). To avoid the high stratification of the subgroups, particularly for both total yield and average fruit weight, we performed a separate GWAS run for each of the subgroups. Using this approach, we identified an additional MTA for average fruit weight (using only the NS subgroup) in a novel location (chromosome 8 12.65 Mb, −log_10_*P* = 5.63; not previously reported according to Vorsa and Zalapa, 2020), where the T allele increases average fruit weight additively by at least 0.5 g (Figure 4D). Fruit rot, anthocyanin content, brix degree, and acids were not as structured as average fruit weight or fruit yield (Figure 4A); therefore, we performed GWAS using all the subgroups combined. For fruit rot, we identified five MTAs (in chromosomes 1, 6, 7, and 11), locations which are consistent with previous QTL studies (Georgi et al., 2013; Daverdin et al., 2017). We did not identify MTA for TACy, Brix, or acids. A list of all MTAs found here is provided on the Supplementary Table 7 for future studies to investigate the co-localization of previously identified QTLs and the MTAs found here.

## Discussion

Cranberry is an iconic species native to North America of great economic, cultural, and traditional value. Despite its recent domestication, cranberry’s evolutionary and genetic history remains mostly obscure. To unravel the cranberry domestication and breeding history, we conducted a genetic diversity study of the largest collection of cranberry accessions in the world, housed at the Rutgers University, Marucci Center for Blueberry and Cranberry Research and Extension Center in Chatsworth, NJ. Cranberry domestication and breeding history were well represented in this germplasm since the collection captures more than 200 years of this crop history. The collection includes native accessions from populations across the species native geographic range, an excellent sample of native selections used in production, and first-, second-, and third-generation hybrids (advanced selections). Importantly, this collection includes accessions from the most important states in the United States, commercially and historically, for cranberry production (Vorsa and Zalapa, 2020). The analyses presented are strongly supported by genome-wide sequence variation revealed by more than 21,000 SNPs, the largest set of markers ever used in cranberry (Figure 1). Compared to previous diversity studies in cranberry (Fajardo et al., 2013; Zalapa et al., 2015; Schlautman et al., 2017a; Schlautman et al., 2018; Rodríguez-Bonilla et al., 2019), both the number of accessions examined and the number of genetic markers are greatly increased.

Our analyses in cranberry, including MAF, population structure (Figure 3A), PCA (Figure 3B), fixation indeces (Figure 2A), ROD estimation (Figure 2B) and linkage disequilibrium decay patterns (Figure 4B), revealed a clear directional and gradual transition from native to modern cultivars/selections, even considering how few breeding and selection cycles have occurred since the first wild selections were made. Particularly, our joint MAF analysis and fixation indeces showed a great differentiation between the start and end points of the breeding/domestication process, WLD versus CLT2 subgroups (highest joint MAF discrepancy between groups, Supplementary Material, and an *F*_ST_ = 0.042, Figure 4A). Between the WLD and CLT2 subgroups there are two centuries, two or three breeding cycles, of cranberry history, which represents a small fraction of time when compared with other crop histories (Meyer et al., 2012); however, during this time period, cranberries have changed considerably both at the genetic and the phenotypic level. Additionally, population structure and PCA helped further investigate how ancestries gradually changed during the breeding process, especially by examining the NS and CLT1 subgroups (Figure 2).

The majority of crops available today, including cereals, fruits, and horticultural species, were derived thousands of years ago (Meyer et al., 2012; Fuller et al., 2012). Cranberry is a very young crop compared to other crop species such as maize (Hufford et al., 2012), rice (Xu et al., 2011), durum wheat (Maccaferri et al., 2019), carrot (Iorizzo et al., 2013), and other fruit crops such as apple (Gross et al., 2014) and peach (Cao et al., 2014). Such species have long domestication histories in which breeding lines and advanced selections significantly contrast, at the genetic and phenotypic levels, from early domesticated forms (Meyer et al., 2012). In these species, both genetic and phenotypic differentiation is the result of the cumulative effect of strong bottleneck events during their long histories (Meyer et al., 2012). Crops domesticated more recently (300 years ago or less) include grapefruit (300 years; de Moraes et al., 2007), African oil palm (200 years; Corley and Tinker, 2008), ackee (200 years; Ekué et al., 2010), dragon fruit (18 years; Tao et al., 2014), macadamia (150 years; Peace et al., 2008), blueberry (100 years; Song and Hancock, 2011) and kiwifruit (50 years; Ferguson, 1984; Li et al., 2014). Particularly for kiwifruit and macadamia, many studies have explored genetic diversity based on collections of early (i.e., wild, first selections) and modern germplasm (i.e., cultivars, advanced selections, elite materials). In kiwifruit, for example, reports show that intensive breeding efforts during the last 20 years have produced today’s great phenotypic diversity, including flesh color, size, metabolite content, etc. (Ferguson and Huang, 2007). However, other studies report that most of the cultivars are direct selections from the wild or seedling populations derived from either *Actinidia chinensis* var. *chinensis* or *A. chinensis* var. *deliciosa* (Ferguson and Seal, 2008), and that despite the high levels of heterozygosity and great diversity on modern cultivated germplasm, they still have a narrow genetic base (Datson and Ferguson, 2011; Li et al., 2014). Similarly, for macadamia, current cultivated trees are a few generations apart from their wild progenitors (Peace et al., 2008; Hardner et al., 2009). Moreover, population structure analysis reveals a continuum in the ancestry proportions among macadamia elite materials, cultivars, hybrids and wild germplasm (Alam et al., 2018), similar to what we observed in cranberry.

Even when comparing crops with similar domestication dates, there are numerous factors that contribute to a rapid or slow diversification, especially when contrasting early and modern germplasm. Major factors shaping the evolutionary history of a crop seem to be related to the high demand or popularity of particular crops worldwide, their annual/perennial nature, and the status of a species prior domestication (Meyer et al., 2012). It is expected that crops like kiwifruit and macadamia, which are comparable to cranberry in terms of the number of generations separating wild and modern materials, were subjected to a more intensive breeding pressure given their worldwide economic importance and cultivar demand, and thus diversified at a larger scale (Hardner et al., 2009; Ferguson and Huang, 2007). Additionally, for perennial fruit crops, domestication is expected to be hampered due to their extended juvenile periods undergone by these crops, which reduce the number of sexual generations, i.e., opportunities for recombination, and the speed of improvement or genetic change (Miller and Gross, 2011; Gaut et al., 2015; Ramu et al., 2017). For example, in blueberry (*Vaccinium corymbosum*), a close relative of cranberry, there is ≤1 chiasma formed per chromosome arm pair during meiosis (Vorsa, 1990), which would produce only 2–3 recombination events per arm, resulting in high linkage disequilibrium in over 100 years. Based on our study, genetic diversity reduction during cranberry domestication and further genetic improvement has not been as pronounced (Figure 4B) as observed in many other crops (Meyer et al., 2012), and this is likely due the very recent history of cranberry domestication along with its growth habit as a long-lived perennial. However, a reduction in diversity is not unexpected since the CLT1 subgroup traces back to only seven native selections (“Early Black,” “Howes,” “McFarlin,” “Searles,” “Potter’s Favorite,” “Prolific,” and “Ben Lear”) and CLT2 back to eight grandparents (same as for CLT1, plus “LeMunyon”). A further reduction in diversity appeared more evident during second breeding cycle selections (cultivar development), which likely corresponded to the selection of fruit quality related traits (i.e., anthocyanin content, Brix, acid, etc.). This is consistent with both wild subgroups and native selections having comparable linkage disequilibrium decay patterns (NS being slightly higher than WLD) (Figure 4B). The status and phenotypic diversity of main target traits during early domestication also affects the evolutionary history of crop species. Fruit size, for example, was one of the main targets during tomato domestication (Lin et al., 2014) and therefore, selection pressure on that trait was intense early in the process. On the other hand, apple domestication and further improvement experienced a lower evolution pressure since its wild progenitor was characterized by larger fruits (Duan et al., 2017).

Based on our genome-wide sequencing polymorphism analysis, there was a clear differentiation between wild and modern cranberry germplasm (Figures 2–4). However, the differences were less clear when comparing phenotypes since most agronomic traits are quantitative. Total yield and average fruit weight clearly differentiated early and modern cranberry forms (Figure 4A). Both the CLT1 and CLT2 had significantly (*P* < 0.01) higher total yield and average fruit weight compared with the other subgroups. On the other hand, except for anthocyanins, fruit metabolite data showed mixed or little differentiation among wild and modern subgroups, indicating neutral selection, reflecting limited directional improvement. For TACy, the only significant (*P* < 0.01) differences were between WLD vs. CLT2 and WLD vs. NS. For Brix degrees, differences were attributable only to CLT2 having significantly higher values than the other three subgroups (*P* = 0.0002 when comparing with CLT1, *P* = 0 when comparing with the rest of the subgroups). A similar tendency was observed for acid, although the differences were more subtle (conversely to Brix, CLT2 had smaller values than the other subgroups). In general, the metabolite accumulation differences between subgroups did not show directional and gradual changes as observed for fruit weight and yield, where modern germplasm are characterized by heavier fruits and more productive varieties. Furthermore, wild and cultivated cranberries still share many morphological similarities and ability to grow well in the same environments. In fact, at the phenotypic level, modern cranberry materials do not exhibit marked differences compared with wild germplasm as in other fruit crops such as kiwifruit (Datson and Ferguson, 2011), a recently domesticated crop, or apple which has a much longer evolutionary history (Spengler, 2019). Thus, despite the clear increases in yield and fruit size in modern materials, the mixed and low differentiation observed among wild and cultivated materials in some traits in our study, likely reflects a recent impetus in improving fruit quality traits such as Brix, acid, and anthocyanin content (Vorsa and Zalapa, 2020).

Initially, early selection from native cranberry populations made in the early 1800’s and continuing for many decades likely selected for larger fruited and productive genotypes, thereby increasing superior alleles for these traits in domesticated varieties. In addition to average fruit weight (size) and total yield, fruit color was also emphasized to some extent (Vorsa and Zalapa, 2020). Based on study, total fruit yield and average fruit weight increased very rapidly in the early stages of breeding. Particularly, CLT1 and CLT2 accessions were characterized by having higher total yield and fruit on average 1 g heavier than WLD and NS germplasm, which jointly with the genetic differentiation quantified here (i.e., WLD and NS vs. CLT1 and CLT2), resulted in a very structured dataset. This stratification, large linkage disequilibrium, made it difficult to identify MTAs even with the use of structure-aware methods, a phenomenon previously investigated (Shin and Lee, 2015). Thus, we conducted an alternative analysis to identify MTA for total yield and fruit weight by making germplasm subsets, in which we identified an additional MTA for average fruit weight using only the NS accessions. This low number of MTA is not surprising since the power to detect marker trait associations using GWAS depends on using large population sizes coupled with ample phenotypic variation in the diversity panel (Jensen et al., 2014). In barley, for example, an association mapping study showed that STRUCTURE-based as well as dimensionality reduction methods (PCA and multidimensional scaling) failed to account for the structure of a highly stratified diversity panel (Wang et al., 2012). Other traits in our study such as anthocyanin content (related to fruit color), Brix degrees, and acid, showed comparable variation among all subgroups; however, we could not identify MTA. We hypothesize that the recurrent introgression of wild and native selections into breeding programs as well as the limited recombination events during cranberry history considerably increased and diversified the causal variants (of potentially low phenotypic effect) observed for these traits, driving up the number of samples required to have enough power to detect an association (Korte and Farlow, 2013; Santure and Garant, 2018). Additionally, simulation studies suggest that statistical power to detect MTAs decreases considerably with the degree of admixture in the population and the use of structure-aware models (Price et al., 2006; Shin and Lee, 2015). For that reason, further attempts to identify MTA through GWAS might require the increase of population sizes (Korte and Farlow, 2013). Additionally, accumulation of anthocyanins, sugars and acids, are very dependent on the ripening stage of the fruit (Vvedenskaya and Vorsa, 2004; Wang et al., 2017). Therefore, quantification of these compounds at multiple time points might be required in order to identify reliable MTAs. Finally, for fruit rot, there was no stratification of the data among the subgroups. In fact, fruit rot was the least differentiated trait among the subgroups. We still identified five MTA (in chromosomes 1, 6, 7, and 11), and some of these MTAs co-localized with some previously identified QTLs (Georgi et al., 2013; Daverdin et al., 2017). For example, both Georgi et al. (2013) and Daverdin et al. (2017) reported fruit rot QTL using four different populations in chromosome 11 in positions 52.13, 57.08, 63.50, and 76.45 cM. These MTA in conjunction with previously detected QTL for fruit rot provide genomic targets for candidate gene discovery and for future breeding efforts to enhance and pyramid disease resistance using marker assisted selection (MAS) (Daverdin et al., 2017).

## Conclusion

This is the first cranberry genetic diversity study involving a very diverse and large collection of germplasm, which captures early domestication events as well as further genetic improvement through breeding. All of the analyses performed here revealed clear pattern of ongoing genetic change characterized by the gradual reduction of wild alleles and transition into elite materials. We hypothesize that the recent domestication of cranberries and the use of recurrent introgression of wild germplasm into cultivars or advanced selections, reduced number of breeding, i.e., recombination, cycles, together with the intricacies of breeding long-lived perennials, has slowed the genetic differentiation between wild and new cranberry materials. Additionally, the wide use of wild germplasm in today’s commercial cranberry production has also likely contributed to slow the implementation of recently released cultivars. The genetic diversity analyses performed in this study collectively revealed important insights into the domestication and breeding history of cranberry and provide a valuable resource for breeding and further genetic improvement of this crop.

## Data Availability Statement

The original contributions presented in the study are publicly available. This data can be found here: doi: 10.6084/m9.figshare.13208360.

## Author Contributions

LD-G, GC-P, NV, and JZ conceived and designed the experiments, revised and approved the final manuscript. NV, JJ-C, and GC-P collected the data. LD-G and JZ analyzed the data and wrote the manuscript. All authors contributed to the article and approved the submitted version.

## Conflict of Interest

The authors declare that this study received funding from Ocean Spray Cranberries, Inc. The funder was not involved in the study design, collection, analysis, interpretation of data, the writing of this article or the decision to submit it for publication.

## Supplementary Material

Supplementary Figure 1 Collection sites of all accessions analyzed on this study.

Supplementary Figure 2 SNP minor allele frequency (MAF) density across the cranberry (Vaccinium macrocarpon Ait.) subpopulations. **(A)** Histograms of MAF across WLD (native populations), NS (native selections), CLT1 (cultivars), CLT2 (advanced selections), and total (all four subpopulations together). **(B)** Joint MAF between all pairs of subpopulations based on two-way incidence tables. Color scale shows the log10 MAF counts using 10 equally spaced bins. Diagonal red lines are showed as reference to illustrate equal categories. Gray colored regions represent joint categories with no counts. Group 1, which is displayed in the x axis, and group 2, which is displayed in the y axis, represent the first and second subpopulations listed on each panel title, respectively.

Supplementary Table 1 Metadata for 362 cranberry accessions.
