# Supplementary figures and images for: Genotyping-by-Sequencing Identifies Historical Breeding Stages of the Recently Domesticated American Cranberry
Source: Front Plant Sci. 2020 Dec 16;11:607770. doi: 10.3389/fpls.2020.607770 (PMC7772218; doi:10.3389/fpls.2020.607770)

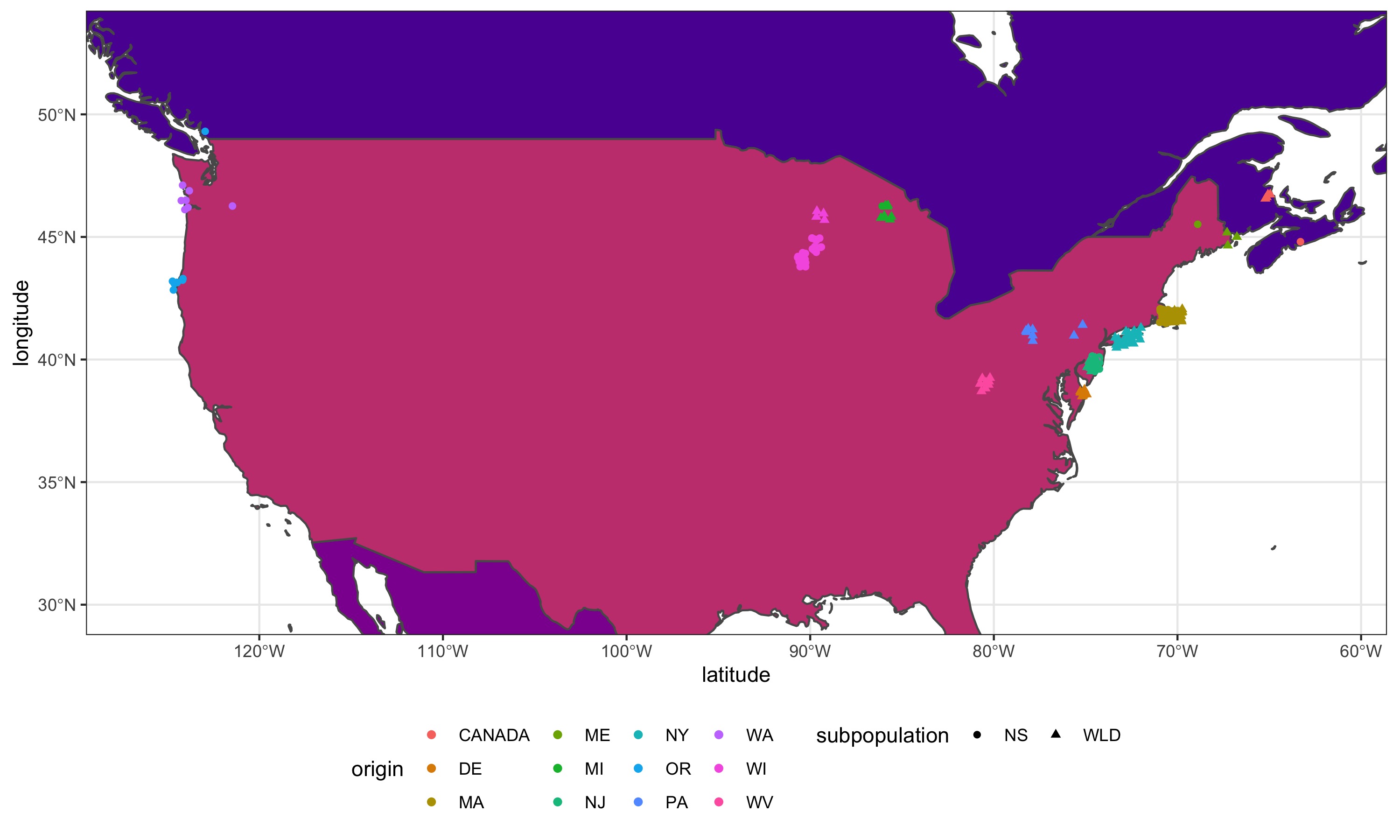

Supplement: Supplementary Figure 1 — Collection sites of all accessions analyzed on this study. [file Image_1.JPEG]

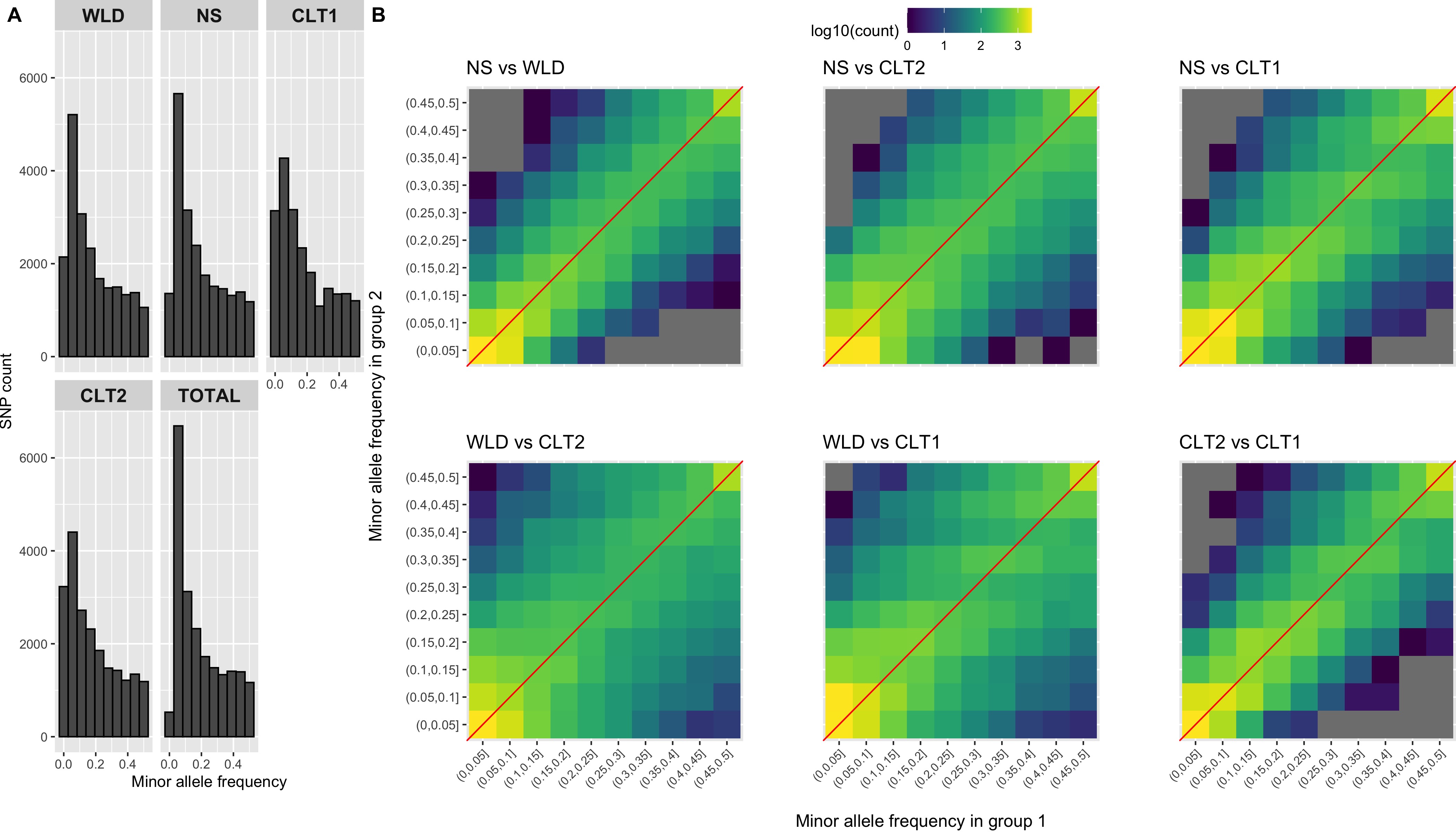

Supplement: Supplementary Figure 2 — SNP minor allele frequency (MAF) density across the cranberry (Vaccinium macrocarpon Ait.) subpopulations. (A) Histograms of MAF across WLD (native populations), NS (native selections), CLT1 (cultivars), CLT2 (advanced selections), and total (all four subpopulations together). (B) Joint MAF between all pairs of subpopulations based on two-way incidence tables. Color scale shows the log10 MAF counts using 10 equally spaced bins. Diagonal red lines are showed as reference to illustrate equal categories. Gray colored regions represent joint categories with no counts. Group 1, which is displayed in the x axis, and group 2, which is displayed in the y axis, represent the first and second subpopulations listed on each panel title, respectively. [file Image_2.JPEG]
